# Effects of Angiotensin II Type 2 Receptor Overexpression on the Growth of Hepatocellular Carcinoma Cells In Vitro and In Vivo

**DOI:** 10.1371/journal.pone.0083754

**Published:** 2013-12-31

**Authors:** Hongyan Du, Zhibing Liang, Yanling Zhang, Feilong Jie, Jinlong Li, Yang Fei, Zhi Huang, Nana Pei, Suihai Wang, Andrew Li, Baihong Chen, Yi Zhang, Colin Sumners, Ming Li, Hongwei Li

**Affiliations:** 1 School of Biotechnology, Southern Medical University, Guangzhou, Guangdong, China; 2 Department of Neuroscience, University of Florida, Gainesville, Florida, United States of America; 3 Department of Pharmacology, University of Florida, Gainesville, Florida, United States of America; 4 Department of Physiology and Functional Genomics, University of Florida, Gainesville, Florida, United States of America; The University of Tennessee Health Science Center, United States of America

## Abstract

Increasing evidence suggests that the renin-angiotensin system (RAS) plays an important role in tumorigenesis. The interaction between Angiotensin II (AngII) and angiotensin type 1 receptor (AT1R) may have a pivotal role in hepatocellular carcinoma (HCC) and therefore, AT1R blocker and angiotensin I-converting enzyme (ACE) inhibitors may have therapeutic potential in the treatment of hepatic cancer. Although the involvement of AT1R has been well explored, the role of the angiotensin II Type 2 receptor (AT2R) in HCC progression remains poorly understood. Thus, the aim of this study was to explore the effects of AT2R overexpression on HCC cells in vitro and in mouse models of human HCC. An AT2R recombinant adenoviral vector (Ad-G-AT2R-EGFP) was transduced into HCC cell lines and orthotopic tumor grafts. The results indicate that the high dose of Ad-G-AT2R-EGFP–induced overexpression of AT2R in transduced HCC cell lines produced apoptosis. AT2R overexpression in SMMC7721 cells inhibited cell proliferation with a significant reduction of S-phase cells and an enrichment of G1-phase cells through changing expression of CDK4 and cyclinD1. The data also indicate that overexpression of AT2R led to apoptosis via cell death signaling pathway that is dependent on activation of p38 MAPK, pJNK, caspase-8 and caspase-3 and inactivation of pp42/44 MAPK (Erk1/2). Finally, we demonstrated that moderately increasing AT2R expression could increase the growth of HCC tumors and the proliferation of HCC cells in vivo. Our findings suggest that AT2R overexpression regulates proliferation of hepatocellular carcinoma cells in vitro and in vivo, and the precise mechanisms of this phenomenon are yet to be fully determined.

## Introduction

Hepatocellular carcinoma (HCC) is one of the most common human cancers worldwide and the third most common cause of cancer-related deaths. More than 80% of HCC cases originate in developing countries [1]. Diagnosis of advanced stage of HCC is a devastating experience for both patients and family. HCC is often diagnosed at an advanced stage when it is no longer susceptible to curative therapies. Moreover, the highly drug-metabolic and multidrug resistant transporter proteins in tumor cells further diminish the efficacy of current therapeutic regimens for HCC [2]. Therefore, alternative approaches are needed to overcome these barriers to enhance therapeutic efficacy.

Gene therapy is a promising treatment for many hereditary diseases such as Leber’s congenital amaurosis, X-linked adrenoleukodystrophy and ‘bubble boy’ disease, and was selected as one of the top 10 breakthroughs of 2009 by the editors of Science. As a major gene therapy milestone, the European Union has recently approved the sale of the Western world’s first gene-therapy drug, Glybera, to treat patients with lipoprotein lipase deficiency [3]. Thus far, most of the clinical trials in gene therapy have been aimed at the treatment of cancer (64.4% of all gene therapy trials). Many different cancers have been targeted throughout the years, including lung, gynaecological, skin, urological, neurological and gastrointestinal tumors, as well as haematological malignancies and paediatric tumors [4]. However, there are currently no approved gene therapy products for cancer in the Western world. Identification of functionally relevant tumor-specific genes for therapeutic targets remains as the major challenge in cancer gene therapy.

Angiotensin II (Ang II), the key effector in the renin-angiotensin system, acts through two well-defined receptors: Ang II type 1 (AT1R) and type 2 receptors (AT2R) [5]. Recent studies suggest that Ang II signaling plays an important role in carcinogenesis [6]–[8]. Using a murine hepatocellular carcinoma development model, Yoshiji and colleagues [9]–[11] showed that combination therapy based on an angiotensin-converting enzyme (ACE) inhibitor (Perindopril [PE]) was able to inhibit angiogenesis mediated by VEGF overexpression. AT2R is known to inhibit cell proliferation and stimulate apoptosis in a variety of cell lines, such as vascular smooth muscle cells, cardiomyocytes, neuronal cells, fibroblasts, endothelial cells, prostate cancer cells and lung cancer cells, but the role of AT2R in HCC progression is currently unclear [12]–[21]. Here we have confirmed the inhibitory effects of adenoviral-induced AT2R overexpression on proliferation and apoptosis of hepatocellular carcinoma cells and have addressed the potential intracellular mechanisms. We have further studied the effects of AT2R on tumor growth in mouse models of the human HCC.

## Materials and Methods

### Cell Cultures

Human hepatocellular carcinoma (HCC) cell lines (SMMC-7721, Bel7402, HepG2) and human fetal liver cell line LO2 were purchased from the Shanghai Institutes for Biological Sciences (China). All cell lines were cultured in Dulbecco’s modified Eagle’s medium supplemented with 10% fetal bovine serum, 100 U/mL penicillin, and 100 µg/mL streptomycin and were maintained at 37°C with 5% CO_2_.

### Recombinant Adenoviral Constructs

For these experiments, we constructed and prepared two recombinant adenoviral vectors as detailed previously [22]: an adenoviral vector containing the enhanced green fluorescent protein gene controlled by a cytomegalovirus promoter (Ad-CMV-EGFP) and an adenoviral vector containing genomic AT2R (G-AT2R) DNA with introns 1 and 2 and the encoding region and enhanced green fluorescent protein gene controlled by cytomegalovirus promoters (Ad-G-AT2R-EGFP).

### Cell Transduction

For viral transduction, HCC cell line cells (3×10^5^) were seeded into six-well Nunc tissue culture plates. On the following day, cells were transduced with Ad-G-AT2R-EGFP or the control vector Ad-CMV-EGFP and changes in cell morphology were observed using an Olympus IX71 fluorescence microscope (Olympus America Inc., PA, USA).

### RNA Isolation and Real-time RT-PCR

HCC cells were cultured in their respective media and harvested for reverse transcription-PCR (RT-PCR). Total RNA for quantitative PCR was isolated with E.Z.N.A.™ total RNA kit I from OMEGA Biotek (Norcross, USA) according to the protocol recommended by the manufacturer. Isolated RNA underwent DNase I (OMEGA Biotek, Norcross, USA) treatment to remove genomic DNA and was then converted into cDNA with Oligo dT and PrimeScript Reverse Transcriptase (TAKARA, Dalian, China) regents. Fluorescent quantitative PCR of AT2R and β-actin was done with SYBR Premix Ex Taq™ (TAKARA) regents. Thermo-cycling conditions were as follows: 95°C for 30 s, followed by 40 cycles of 5 s at 95°C and 34 s at 60°C. The oligonucleotide sequences of forward and reverse primers used for endogenous AT2R were as follows: forward, 5′-CGGAATTCATGAGCTGCGTTAATCC-3′; reverse 5′-AACTGCAGTTAAGACACAAAGGTCTCCA-3′ and the primers for AT2R overexpression were as follows: forward, 5′-CCGCATTTAACTGCTCACACA-3′; reverse 5′-ATCATGTAGTAGAGAACAGGAATTGCTT-3′. The samples were analyzed with the ABI 7500 real-time PCR system (Applied Biosystems) and subjected to comparative △△Ct method by using human β-actin as the internal standard. Real-time PCR products (8 µL) were loaded on 2.5% agarose gel containing ethidium bromide.

### Cell Cycle Analyses

For cell cycle analysis, samples (1×10^6^ cells) were fixed and permeabilized by addition of 1 mL of ice-cold 70% ethanol for 2 hrs at 4°C. After washing, the cells were treated with KeyGEN Cell Cycle Detection Kit (KeyGEN Biotech, Nanjing, China) following the protocol. The cells were resuspended in 100 µL RNase A and incubated at 37°C for 30 min. Next, 400 µL propidium iodide was added to the cells. Following treatment for 30 min at room temperature in the dark, the cells were stored at 4°C until analysis by flow cytometry (FACSCalibur, BD Biosciences). Cell cycle analysis was done using ModFit LT software (Verity).

### Cell Proliferation and Cytotoxicity Assays

For Cell Proliferation and cytotoxicity Assays, samples (5×10^3^ cells) were placed into 96-well plate. Cell proliferation and cytotoxicity were evaluated using a WST-1 Cell Proliferation and Cytotoxicity Assay Kit (Beyotime Institute of Biotechnology, Jiangsu, China). WST-1 reagent was added to the culture medium (1∶10 dilution), and absorbance was measured at 450 nm with Varioskan Flash microplate reader (Thermo Fisher Scientific, Waltham, MA).

### Apoptosis Assays

Apoptosis of viral vector-transduced cells was measured using a DeadEnd Colorimetric terminal deoxynucleotidyl transferase-mediated dUTP nick end labeling (TUNEL) System (Promega) and One Step TUNEL Apoptosis Assay Kit (Beyotime). At 24 hrs after 500 ifu/cell viral vector transduction, the growth medium was aspirated, and cells were fixed with 4% formaldehyde in PBS (pH 7.4) for 25 min at room temperature, then washed twice for 5 min in PBS, permeabilized in 0.2% Triton X-100 solution in PBS for 5 min at room temperature, and finally washed twice for 5 min in PBS. DeadEnd Colorimetric TUNEL System was used according to the manufacturer’s instructions. Cells were mounted in Vectashield +4′, 6-diamidino-2- phenylindole (Vector Labs) to stain nuclei. The number of TUNEL-positive cells (brown color) in each treatment condition was counted from 10 randomly selected fields per well by an individual who was blinded as to the treatment. Data are presented as a percent of the total number of cells on the dish, which was assessed from 4′, 6-diamidino-2-phenylindole staining. The number of stained cells that exhibit apoptotic-like morphology was assessed by counting cells from 10 randomly chosen fields per well.

### Western Blotting

Western immunoblots were performed as described previously [14]. Primary antibodies including anti-caspase3, anti-activated caspase 3, anti-activated caspase 8, anti-total p38 MAPK, anti- Phospho-p38 (pp38) MAPK, anti-total p44/42 MAPK (Erk1/2), anti-Phospho-p44/42 MAPK (Erk1/2), anti-total SAPK/JNK, anti- Phospho-SAPK/JNK (pJNK), anti-PP2A, anti-CDK4, anti-cyclin D1 and anti-β-actin were from Cell Signaling Technology. Anti-GAPDH was from Bioworld Technology, Inc. The secondary antibodies horseradish peroxidase–conjugated anti-rabbit IgG and anti-mouse IgG were from Beyotime Institute of Biotechnology.

### Caspase-3 Like Protease Activity

Caspase-3-like protease activity was assessed using the Caspase 3 Activity Assay Kit (Beyotime). Standard curve was made first using standard sample pNA from the assay kit. Transduced and control cells (10^6^) were lysed in the lysis buffer provided by the kit followed by centrifugation (16,000×g for 15 min at 4°C). Caspase-3-like activity was assessed in supernatants by following the proteolytic cleavage of the colorimetric substrate Ac-DEVD-ρNA. Samples, total volume 100 µL, were read at 405 nm in a Varioskan Flash microplate reader (Thermo Fisher Scientific, Waltham, MA) using an ELISA plate.

### Intrahepatic Tumor Model

All procedures were performed in accordance with the guidelines and approval of the local Institutional Animal Experimentation Ethics Committee. BALB/c nude mice of 4 to 5 weeks of age were purchased from the Experimental Animal Center of the Guangzhou University of Traditional Chinese Medicine (China) and were maintained under standard pathogen-free conditions. Mice were anaesthetized by chloralic hydras (3.5%). SMMC7721 cells (2.5×10^6^), transfected with a lentivirus vector containing CMV-driven luciferase gene, in 50 ul PBS were orthotopically injected into liver of each BALB/c nude mouse during laparotomy. The injection site was compressed for 1 min to control bleeding, followed by closure of the laparotomy. After three days, a dose of 10^9^ infectious units (ifu) of the viruses (Ad-CMV-EGFP or Ad-G-AT2R-EGFP) suspended in 100 µL of PBS or 100 µL of PBS alone was administrated via injection into the tail vein every two days. Total 3 administrations were performed for each mouse. Each group consisted of 8 mice. The mice were euthanized 4 weeks later. Liver tumors in situ were collected, weighted, photographed, and then preserved in liquid nitrogen for further study. The expressions of AT2R in liver tissue were detected using real time RT-PCR.

### Bioluminescence Image Analysis

The growth and metastasis of the HCC cell SMMC7721 tumors in the mouse were monitored using Xenogen IVIS-200 Optical in Vivo Imaging System as described previously [23], [24]. Light was monitored in all of the experiments described at 5 min after injection of luciferin. The CCCD signals were quantified as total relative light units per minute of acquisition time (RLU/min) in the region of interest (ROI).

### Immunohistochemistry

Tumor tissues were fixed in cold 4% paraformaldehyde for 24 hr and then embedded in paraffin blocks. Afterward the paraffin blocks were cut into 5-µm sections, which were stained with hematoxylin and eosin (H&E) to determine morphology. To determine cell proliferation in the tumors, the expression of Ki67 in liver tumors was examined by Immunostain SP Kit (Zhongshan Gene Bridge Biotechnology Company, Beijing, China). The anti-Ki67 primary antibody was obtained from Millipore (Merck KGaA, Darmstadt, Germany) and PBS instead of Anti-Ki67 primary antibody as negative control. Immunohistochemistry stained samples were assessed by two researchers blinded to the samples. The final staining score was presented according to previous method [25], [26].

### Data Analysis

Unless otherwise specified, data are representative of at least three independent experiments and expressed as mean ± SE. Statistical significance was calculated by two-tailed Student’s t-test and p<0.05 or p<0.01 was considered significant.

## Results

### Endogenous and Adenoviral-Mediated Expression of AT2R in HCC Cells

The presence of endogenous AT2R in human HCC cells was detected by real-time PCR. Endogenic AT2R mRNA expression was minimally detectable in basal HCC cell lines. Ct values for endogenic AT2R mRNA in untreated SMMC7721, Bel7402, or HepG2 cells were all greater than 33.0, which are defined as negative range (data not shown).

**Figure 1:**
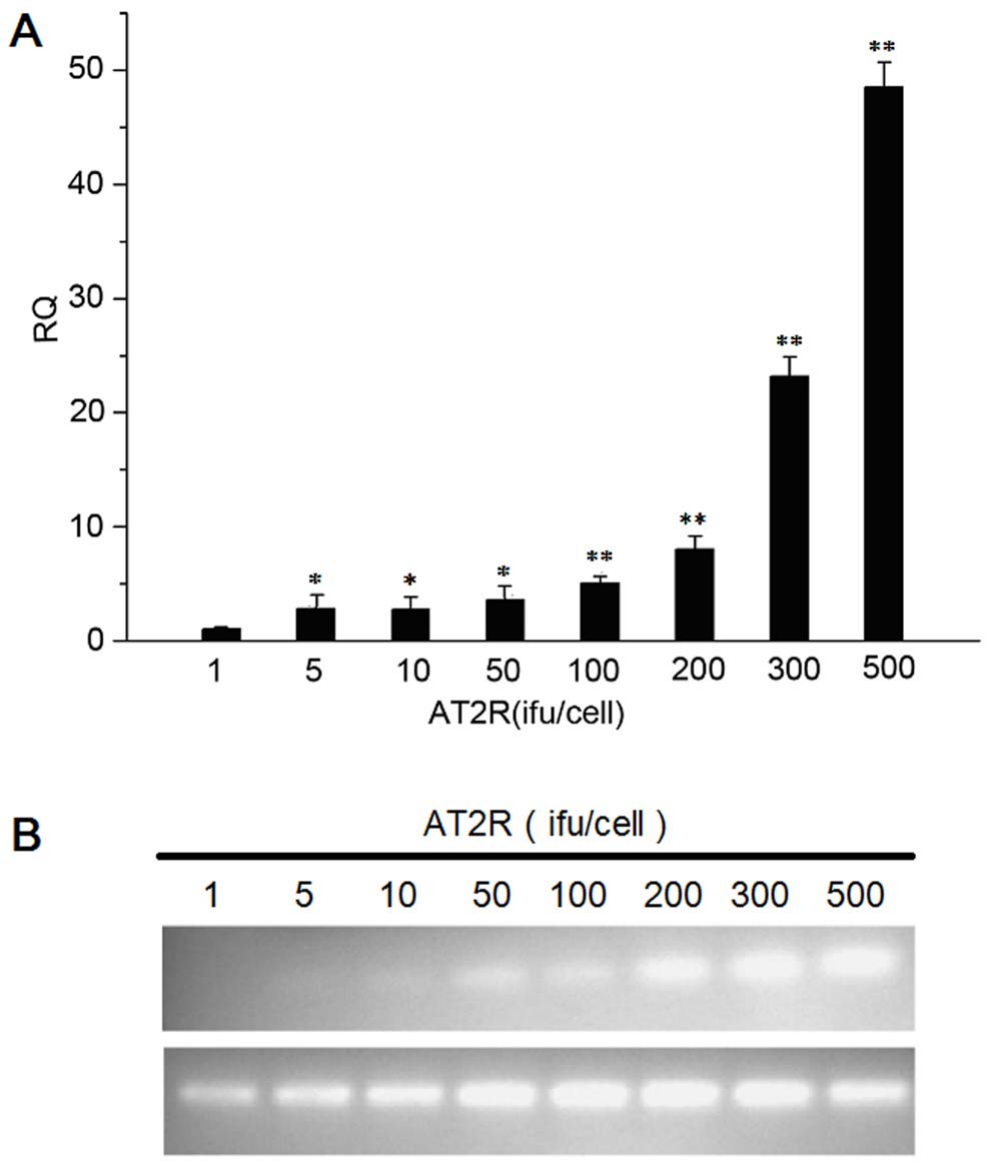
Basal and viral vector–mediated expression of AT2R in HCC line SMMC7721. **A**, RQ comparison of AT2R mRNA expression following transduction with Ad-G-AT2R-EGFP gradient doses (1 ifu, 5 ifu, 10 ifu, 50 ifu, 100 ifu, 200 ifu, 300 infu, 500 ifu/cell). Columns, mean (n = 3); *P<0.05 and **P<0.01 versus 1 ifu of Ad-G-AT2R-EGFP–transduced cells. **B**, ethidium bromide-stained gels show AT2R and β-actin mRNA expression in transduced SMMC7721 cells.

SMMC7721 cells were then transduced with either gradient doses of Ad-G-AT2R-EGFP or the control vector Ad-CMV-EGFP (1 ifu, 5 ifu, 10 ifu, 50 ifu, 100 ifu, 200 ifu, 300 ifu, 500 ifu/cell). Total RNA was extracted, and AT2Rs were detected by real-time PCR. The relative expression quantity (RQ) of AT2R following AT2R (1–500 ifu/cell) transduction were showed in Fig. 1A and B. Data indicated an AT2R overexpression in SMMC7721 cells with Ad-G-AT2R-EGFP transduction, consistent with previous report [20]. Similar results were obtained following Ad-G-AT2R-EGFP infection of Bel7402 and HepG2 HCC cells and LO2 human fetal liver cell line (data not shown).

### The Effect of AT2R on HCC Cell Growth and Cycle

**Figure 2:**
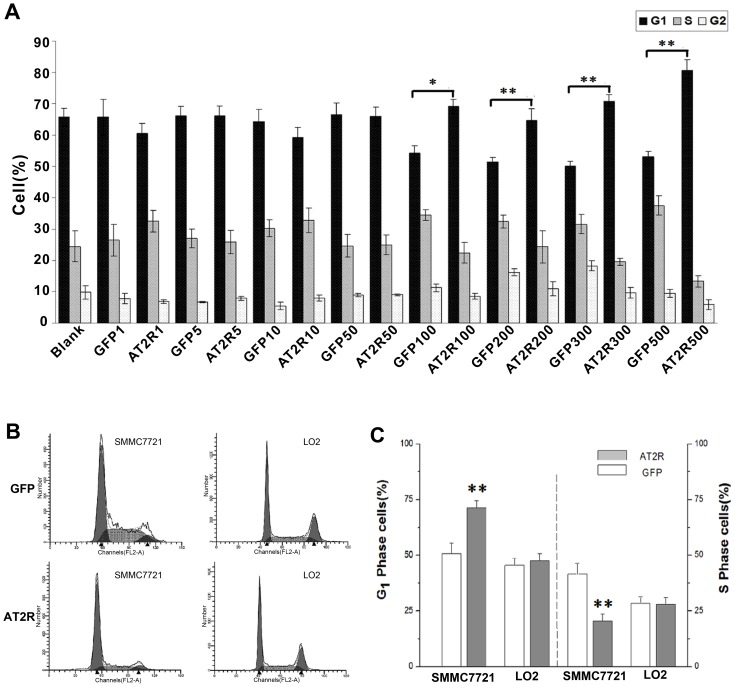
AT2R overexpression disturbs cell cycle in SMMC7721 but not LO2 cells. **A**, the percentages of SMMC7721 cells transduced with 1–500 ifu/cell of either Ad-G-AT2R-EGFP or Ad-CMV-EGFP for 24 hrs in G1 phase, S phase and G2 phase. Columns, mean (n = 3); bars, SE. Data are derived from DNA histograms.*P<0.05 and **P<0.01 versus Ad-CMV-EGFP–transduced cells. **B**, fluorescence intensity of SMMC7721 and LO2 cells transduced with 300 ifu/cell of either Ad-G-AT2R-EGFP or Ad-CMV-EGFP for 24 hrs was measured by flow cytometry. **C**, data showing percentages of SMMC7721 and LO2 cells in G1 phase (left) and S phase (right) under each treatment condition. Columns, mean (n = 3); bars, SE. Data are derived from DNA histograms. **P<0.01 versus Ad-CMV-EGFP–transduced cells.

**Figure 3:**
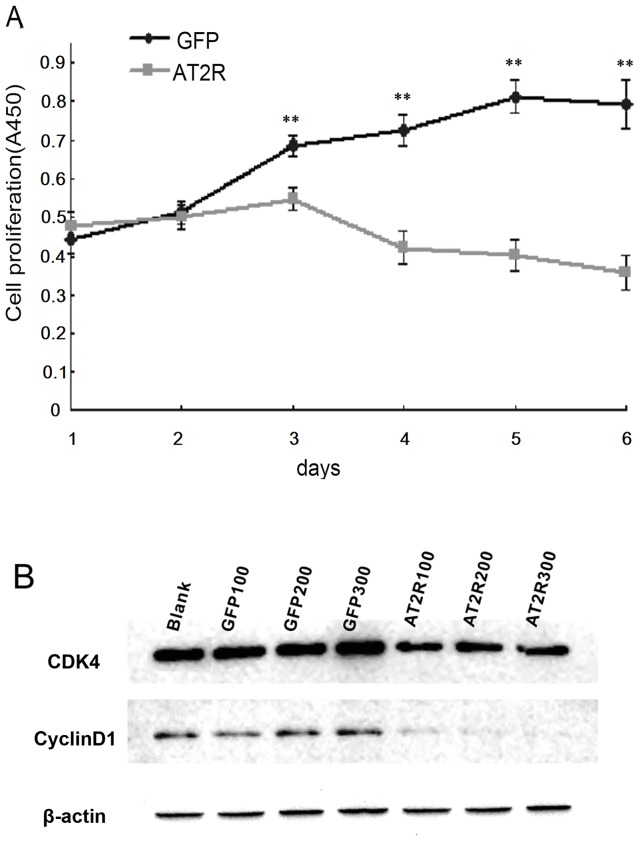
AT2R overexpression inhibits cell proliferation in SMMC7721 cells. **A,** growth curve of HCC SMMC7721 cells with 200/cell of Ad-G-AT2R-EGFP and Ad-CMV-EGFP–transduced. **P<0.01. **B**, representative Western blots showing the CDK4 and cyclin D1 in SMMC7721 cells induced for 24 hrs with Ad-G-AT2R-EGFP and Ad-CMV-EGFP (100, 200 and 300 ifu/cell, respectively). Data are representative of three experiments.

HCC SMMC7721 cells were transduced with 1–500 ifu/cell gradient concentrations of either Ad-G-AT2R-EGFP or Ad-CMV-EGFP for 24 hrs, then fixed, permeabilized with ethanol, and stained with propidium iodide. The cell cycle profiles were examined by FACS as described in Materials and Methods. Transduction SMMC7721 cells with Ad-G-AT2R-EGFP (100–500 ifu/cell, 24 hrs) produced a significant reduction in the number of S-phase cells and an increase in G1-phase cells as compared to the cells transduced with Ad-CMV-EGFP (100–500 ifu/cell, 24 hrs). No significant changes in the cell cycle profiles were observed in human fetal liver LO2 cells (Fig. 2A, B and C). SMMC7721 cells treated with Ad-G-AT2R-EGFP also demonstrated significantly attenuated proliferation (Fig. 3A). To illuminate the possible mechanism(s) underlying AT2R-mediated inhibition of cell growth, key components in cell cycle control such as CDK4 and cyclinD1 were analyzed. The expressions of CDK4 and cyclinD4 in SMMC7721 cells received 100–300 ifu/cell of Ad-G-AT2R-EGFP were down-regulated relative to that in cells treated with Ad-CMV-EGFP (Fig. 3B). These results suggest that AT2R overexpression in SMMC7721 cells inhibits cell proliferation with a significant reduction of S-phase cells and an enrichment of G1-phase cells through changing expressions of CDK4 and cyclinD1.

### AT2R Inducing Apoptosis in HCC Cells

**Figure 4:**
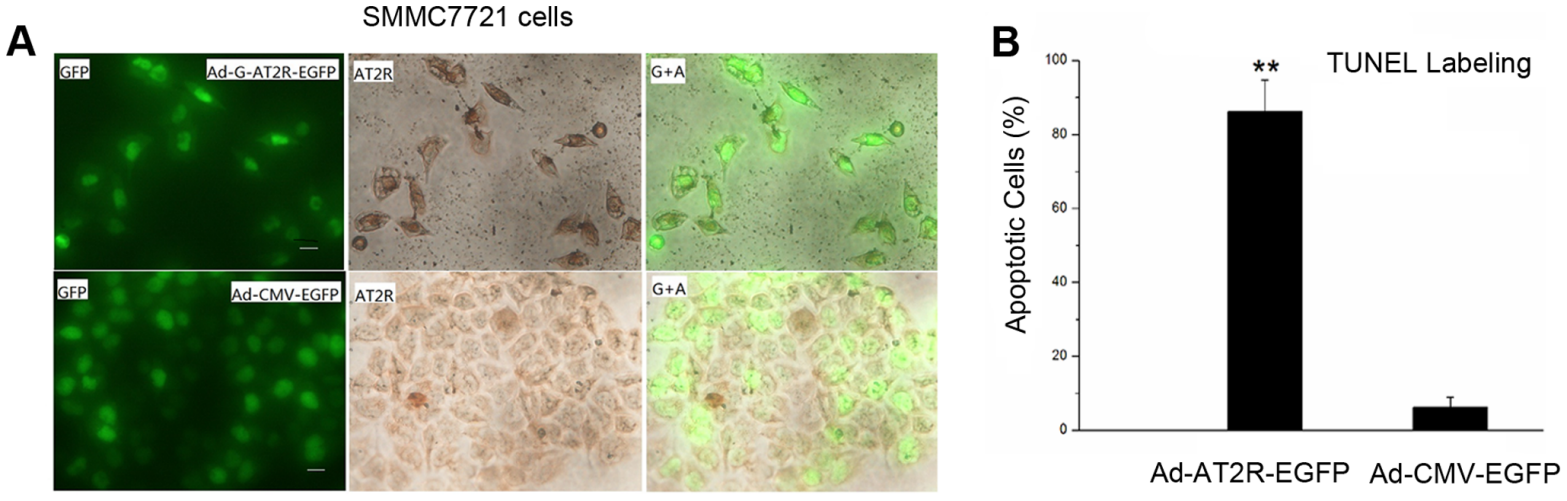
AT2R overexpression induces apoptosis of SMMC7721 cells. **A**, SMMC7721 cells were transduced with either Ad-CMV-EGFP or Ad-G-AT2R-EGFP (500 ifu/cell) for 24 hrs as described in materials and methods and apoptotic cells were detected using the DeadEnd Colorimetric TUNEL. Two representative phase-contrast micrographs from each treatment condition. Representative fluorescence micrographs from Ad-CMV-EGFP–transduced and Ad-G-AT2R-EGFP–transduced cells, showing EGFP fluorescence, TUNEL-positive (apoptotic) cell (brown-colored nuclei), and merged (G+A) EGFP/AT2R in each treatment condition. *Scale bars*, 50 µm. **B**, quantification of the TUNEL-positive cells as a percent of the total number of cells in the dish. Columns, mean of three experiments; bars, SE. **P<0.01 versus GFP group.

Transduction of SMMC7721 cells and Bel7402 with Ad-G-AT2R-EGFP (500 ifu/cell) for 24 hrs resulted in a large number of cells that exhibited apoptotic-like morphologic changes, including irregular-shaped nuclei and a clear boundary between nuclei and cytoplasm, as compared with the controls (Fig. 4A and Fig.S1A). Similar treatment of HepG2 cells with Ad-G-AT2R-EGFP also resulted in the appearance of apoptotic-like morphology in some cells, although the effect appeared to be weaker than that observed in SMMC7721 and Bel7402 cells (data not shown).

Because this effect of AT2R was significant and easily recognizable in SMMC7721 cells, our subsequent experiments were mostly focused on this tumor cell line. The apoptotic action following AT2R transduction was confirmed by the finding that incubation of SMMC7721 cells with Ad-G-AT2R-EGFP (500 ifu/cell) for 24 hrs produced a significant increase in TUNEL labeling compared with the Ad-CMV-EGFP (500 ifu/cell)–treated cells. In contrast to the HCC cells, human fetal liver LO2 cells transduced with Ad-G-AT2R-EGFP (500 ifu/cell) for 24 hrs exhibited no significant morphologic changes as compared to LO2 cells with Ad-CMV-EGFP–transducion (data not shown). Consistent with these findings, AT2R overexpression in HCC cell cultures caused significant cytotoxicity, whereas no cytotoxic effect was observed in LO2 cells. Specifically, SMMC7721 and Bel7402 cultures displayed 83.2±6.2% and 79.3±8.8% cell apoptosis (n = 3 experiments) post Ad-G-AT2R-EGFP transduction as compared to 4.9±0.7% and 5.8±0.6% cell apoptosis (n = 3 experiments) in Ad-CMV-EGFP–transduced cultures (Fig. 4B and Fig.S1B). In comparison, the levels of cell death in Ad-GAT2R-EGFP–transduced and Ad-CMV-EGFP–transduced LO2 cultures were 3.7±0.8% and 3.9±0.5%, respectively (n = 3 experiments).

### AT2R-induced HCC Cell Apoptosis is Mediated by MAPK Pathway and Caspase-3 and Caspase-8

**Figure 5:**
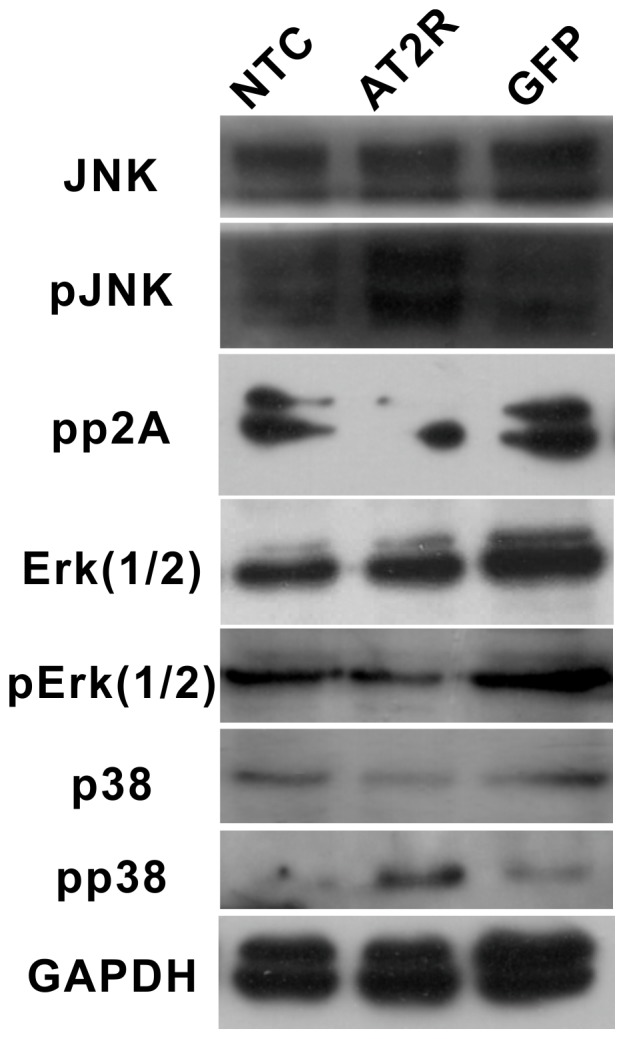
Involvement of MAPK superfamily in AT2R-induced apoptosis in SMMC7721 HCC cells. Representative Western blots show the p38 MAPK and pp38 MAPK in SMMC7721 cells transduced for 12-G-AT2R-EGFP and Ad-CMV-EGFP (500 ifu/cell) or mock-transduced and PP2A, JNK, pJNK, p42/44 MAPK(Erk1/2) and pp42/44 MAPK(pErk1/2) in SMMC7721 cells transduced for 24 hrs. Data are representative of three experiments.

HCC SMMC7721 cells transduced with Ad-G-AT2R-EGFP, Ad-CMV-EGFP and with control mock-transduction were analyzed for MAPK superfamily proteins. Immunoblot analysis indicated the presence of a ∼40 kDa and 57 kDa protein that cross-reacted with a specific antibody against activated p38 MAPK (pp38) and pJNK in SMMC7721 cells (Fig. 5). Moreover, the Ad-G-AT2R-EGFP treatment increased basal levels of pp38 MAPK and pJNK in a dose independent manner as compared to Ad-CMV-EGFP–treated or mock-transduced SMMC7721 cells. Basal levels of total p38 MAPK and JNK were unchanged under these treatment conditions (Fig. 5). Meanwhile, it was found that pp42/44 MAPK (Erk1/2) and its upstream protein, PP2A were also attenuated in SMMC7721 cells following Ad-G-AT2R-EGFP treatment. The activation of class II caspases is considered a hallmark of programmed cell death [27]. For example, caspase-8 is required for the initiation phase of the extrinsic cell death signaling pathway, whereas activation of caspase-9 is integral to the intrinsic cell death pathway that involves mitochondrial release of cytochrome C [28].

**Figure 6:**
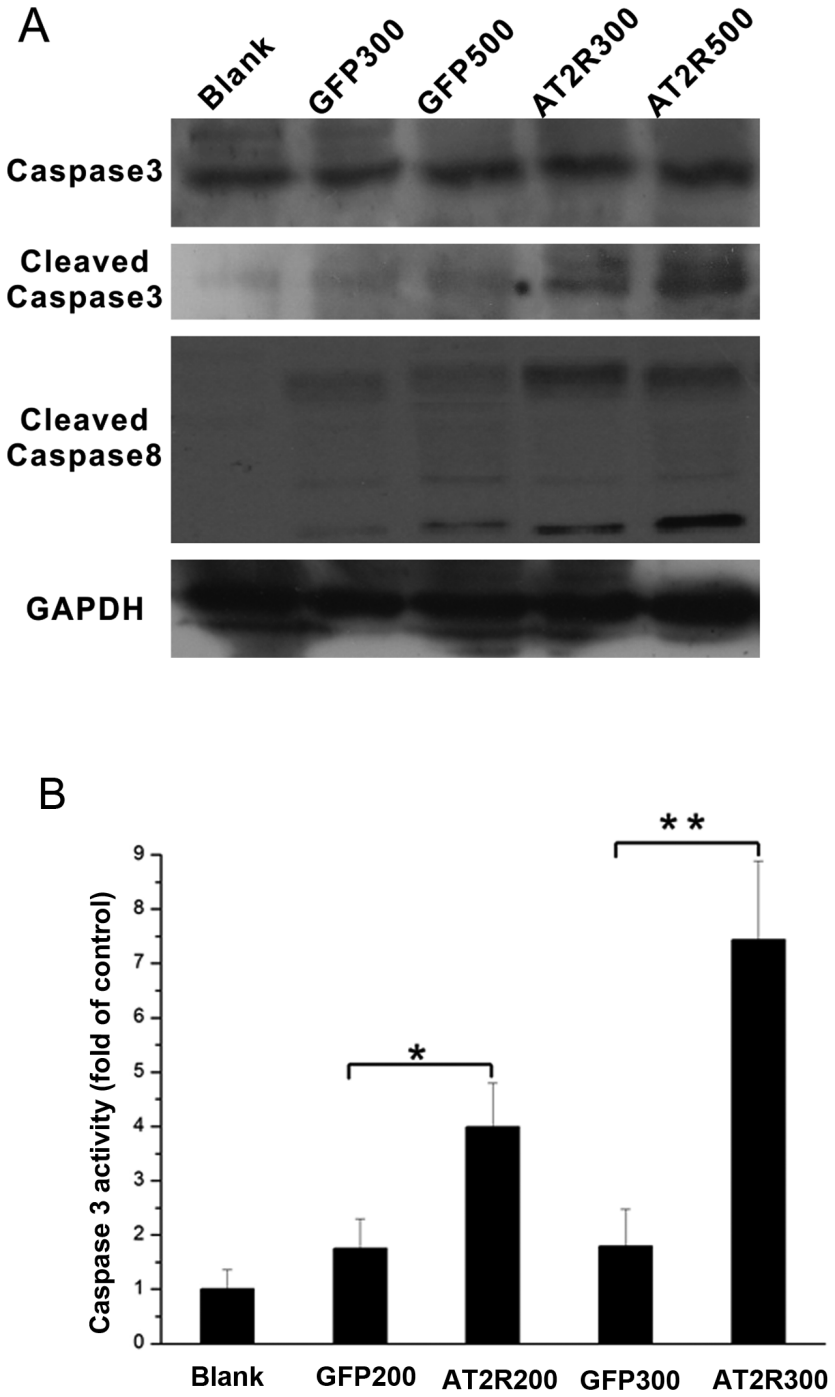
Role of caspases in AT2R-induced apoptosis in SMMC7721 HCC cells. **A,** Western blot analysis of AT2R-induced increase in cleaved caspase-3 and caspase-8 generation in SMMC7721 cells. Cells were infected after 24 hrs with either 300 or 500 ifu/cell of Ad-CMV-EGFP, Ad-G-AT2R-EGFP or mock-transduced. Data are representative of three experiments. **B**, AT2R-induced increase in caspase-3 activity in SMMC7721 cells. Columns, mean A 405 nm from three experiments; bars, SE. *P<0.05 and **P<0.01 versus Ad-CMV-EGFP–transduced or mock-transduced cells.

Initiator caspase activation leads to the eventual proteolytic activation of the effector caspase-3, which cleaves a large number of cellular proteins leading to apoptosis. We first examined the role of caspase-3 in apoptosis induced by overexpression of AT2R. Cell extracts prepared from Ad-G-AT2R-EGFP (500 ifu/cell)–transduced SMMC7721 cells displayed significantly higher caspase-3-like activity compared with the extracts from Ad-CMV-EGFP (500 ifu/cell)–transduced cells or mock-transduced cells. Ad-G-AT2R-EGFP (500 ifu/cell) treatment in SMMC7721 cells resulted in cleavage of the ∼32-kDa caspase-3 protein to yield the active ∼17- and 19-kDa subunits and cleavage of the caspase-8 protein to yield the active∼18-and 43-kDa subunits as shown by Western blot (Fig. 6A). When assayed for caspase-3–like activity via measurement of the release of ρNA from the colorimetric caspase-3 substrate z-DEVD-ρNA, caspase-3 activities were significantly up-regulated in SMMC7721 cells received Ad-G-AT2R-EGFP as compared to controls (Fig. 6B). Collectively, these data suggest that AT2R induction of HCC cells apoptosis impacts distinct members of the MAPK superfamily and involves a caspase-8-mediated extrinsic signaling pathway followed by downstream activation of caspase-3 in mechanism.

### Effects of AT2R on the Growth of HCC Cells in Vivo

**Figure 7:**
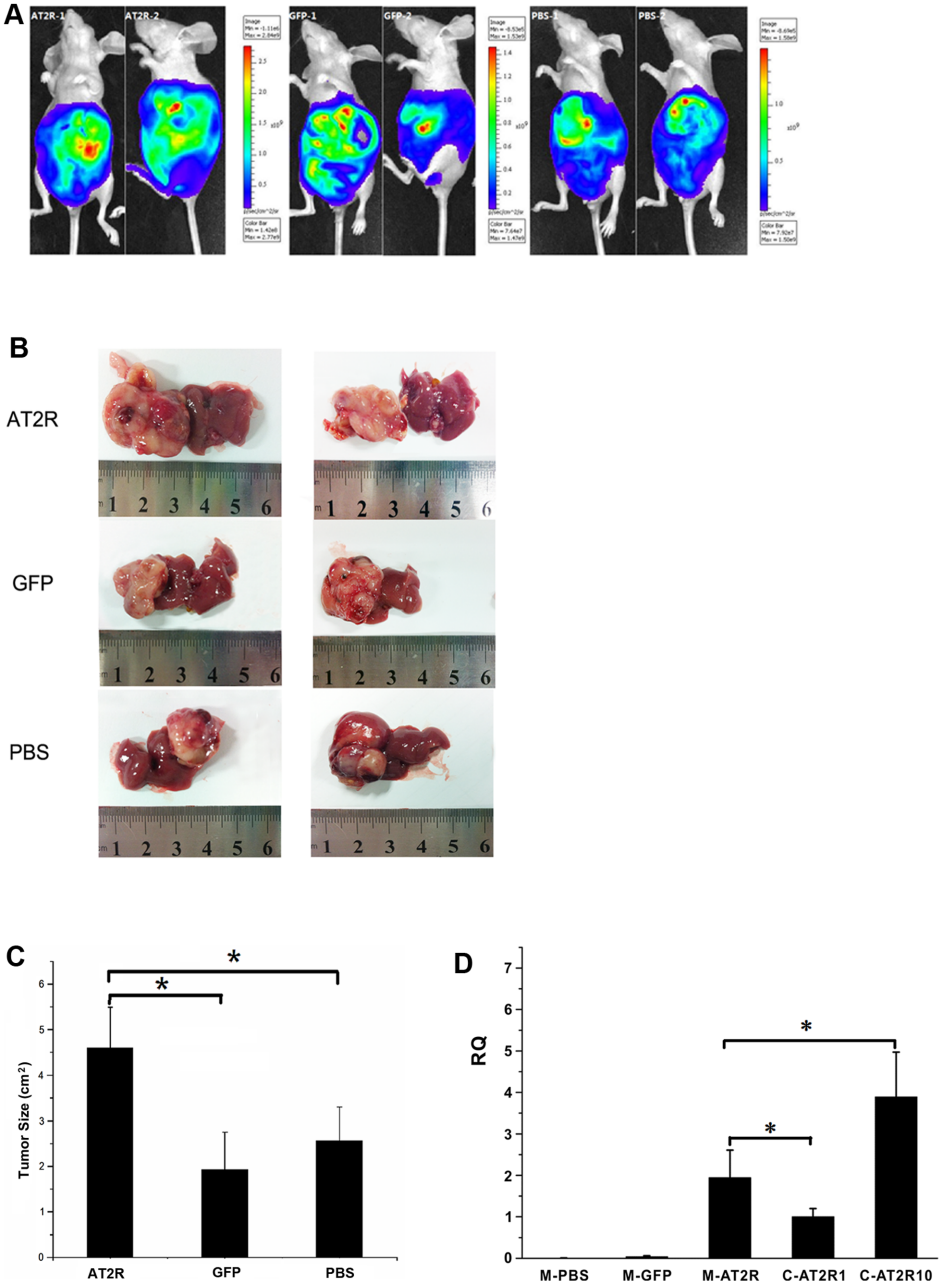
The effect of AT2R overexpression on the growth of HCC tumors. **A**, CCCD signals of cellular orthotopic injection animal models post transfection with Ad-CMV-EGFP, Ad-G-AT2R-EGFP tail vein injection and PBS as negative control. **B**, liver tumor tissues were separated from human hepatocellular carcinoma nude mice models. **C**, the effect of AT2R on tumor size. Data are expressed as mean ± SD. (*P<0.05, n = 8). **D**, RQ comparison of AT2R mRNA expression in animal models (M-PBS; M-GFP; M-AT2R) and SMMC7721 cells. M-AT2R, M-GFP and M-PBS, AT2R mRNA expression in liver tumor tissues of the nude models injected with Ad-G-AT2R-EGFP, Ad-CMV-EGFP, PBS. C-AT2R1 and C-AT2R10, AT2R mRNA expression in SMMC7721 cells following transduction with gradient doses of Ad-G-AT2R-EGFP (1ifu, 10 ifu/cell). Columns, mean (n = 3); *P<0.05 versus 1 and 10 ifu/cell of Ad-G-AT2R-EGFP–transduced cells.

**Figure 8:**
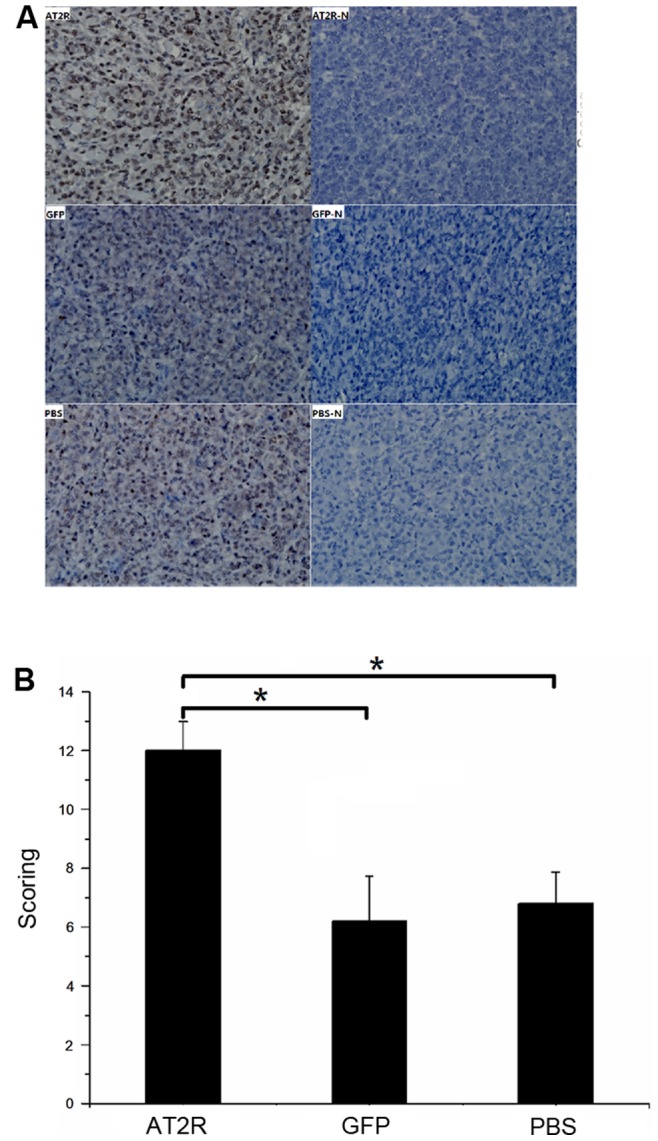
The effect of AT2R overexpression on Ki67 expression in vivo. **A**, the Ki67 immunohistochemistry staining of liver tumor tissues from human hepatocellular carcinoma nude mouse models injected with Ad-CMV-EGFP, Ad-G-AT2R-EGFP or PBS via tail vein. AT2R-N, GFP-N and PBS-N, PBS instead of Anti-Ki67 primary antibody as negative control (×400). **B**, the immunohistochemistry staining scoring from different groups. Data are expressed as mean ± SD. (*P<0.05, n = 8).

To determine whether AT2R regulates the growth of HCC tumors in vivo, we employed a cellular orthotopic animal model of BALB/c nude mice. SMMC7721 cells expressing luciferase were injected orthotopically into the liver of these mice. Afterward D-Luciferin was injected as a substrate, and the animals were anesthetized and bioluminescence image analysis was carried out. The CCCD camera was used to monitor luciferase gene expression in the mouse model of HCC. CCCD signals were quantified as p/sec/cm^2^/sr. The bioluminescence signal of Ad-G-AT2R-EGFP treatment group ranging from 1.11×10^6^ to 2.84×10^9^ p/sec/cm^2^/sr, was significantly higher than in Ad-CMV-EGFP treatment group and PBS group, ranging from 8.53×10^5^ to 1.53×10^9^ and 8.69×10^5^ to 1.58×10^9^ p/sec/cm^2^/sr(n = 8) (Fig. 7A). Furthermore, Ad-G-AT2R-EGFP treatment significantly increased the tumor growth of SMMC-7721 in comparison with Ad-CMV-EGFP and PBS control (n = 8, p<0.05) (Fig. 7B and C). We next detected the AT2R expression in tumor tissues using real-time PCR and found that the level of AT2R mRNA in Ad-G-AT2R-EGFP treatment group was significantly lower than that in SMMC-7721 cells transduced with 10 ifu/cell of Ad-G-AT2R-EGFP (Fig. 7D). AT2R mRNA expression was undetectable in either the Ad-CMV-EGFP or PBS group. Immunohistochemistry analysis was subsequently performed to examine Ki67 (an anti–proliferating cell nuclear antigen) expression in HCC tumor tissues. As shown in Fig. 8, the Ad-G-AT2R-EGFP treatment in mice resulted in significantly enhanced SMMC-7721 cell proliferation, opposing what was observed with SMMC-7721 cell culture model of high dose Ad-G-AT2R-EGFP transduction (P<0.05). Taken together, these data implicate that whereas high level of overexpression of AT2R may induce apoptosis and inhibit tumor cell proliferation, moderately increasing AT2R expression in vivo may actually promote HCC SMMC7721 cell proliferation and the tumor growth.

## Discussion

In the present study, we first showed that endogenic AT2R mRNA expression was minimal in HCC SMMC7721, Bel7402 and HepG2 cell lines. High level of AT2R overexpression in SMMC7721 cells reduced S-phase cells and enhanced G1-phase cells via a suppression of CDK4 and cyclinD1 expression and ultimately inhibited cell proliferation. However, the lower dose of Ad-G-AT2R-EGFP didn’t affect cell proliferation and cell cycle (Fig. 2 and Fig. 3). Our data also demonstrate that the high Ad-G-AT2R-EGFP dose –induced AT2R overexpression in all the HCC cell lines tested produced apoptosis (Fig. 4) involving cell death signaling pathway that is dependent on activation of p38 MAPK, pJNK, and caspase-3 and inactivation of pp42/44 MAPK (Erk1/2) (Fig. 5 and Fig. 6). Collectively, these findings illustrate the ability of increased AT2R expression to induce apoptosis and inhibit tumor cell proliferation in vitro.

AT2R has been shown to promote apoptosis in a variety of cell lines [12]–[14], [18]–[22]. Gene transfer-mediated AT2R overexpression constitutively induces apoptosis in prostate cancer cell lines [20], lung cancer cell lines [21], cardiomyocyte [12], [14] and vascular smooth muscle cells [29]. On the contrary, increased level of the AT2R has been indicated to lead to ligand-independent, constitutive cardiomyocyte hypertrophy [30]. There was the notion suggesting that high level of AT2R causes LV dysfunction and myocyte apoptosis [12], [15]. Consistent with the earlier reports [12], [15], high level of AT2R elevated apoptosis in HCC cell lines in the present study, while moderate and low level of AT2R did not impact apoptosis. This may suggest that the level of AT2R expression modulates the different effects of AT2R on the cells.

Recombinant adenoviral-mediated AT2R gene transfer is becoming a powerful tool to investigate AT2R functions in various cell types. It significantly increases AT2R expression in a multitude of cell lines such as C2C12 mouse muscle myoblasts, NIH/3T3 fibroblasts, CATH.a [22], human prostate cancer cell lines DU145, LNCaP and PC3 [20], primary cultures of neonatal rat cardiac fibroblasts and cardiomyocyte [12], [31]. Our data also prove that Ad-G-AT2R-EGFP effectively enhanced AT2R expression in HCC cells.

Apoptosis is an important mechanism by which cancer therapeutic agents induce cancer cell death. AT2R-mediated apoptosis seems to may involve different cellular mechanisms of apoptosis depending on the cell type. In intestinal epithelial cells, for example, Ang II signals through the AT2R to up-regulate GATA-6 expression, which in turn up-regulates the expression of Bax, eventually leading to apoptosis in these cells [13]. In HL-1 cardiomyocytes, on the other hand, iNOS upregulation following forced AT2R expression seems to be the basis for an increase in cardiomyocyte apoptosis [14]. In another scenario, AT2R signaling stimulates a MAPK tyrosine phosphatase, (MKP)-1, which inhibits MAPK activation and consequentially inactivates Bcl-2 and induces apoptosis in proximal tubular cells [16]. Apoptotic cell death induced by AT2R overexpression in R3T3 fibroblasts, Chinese hamster ovary epithelial cells, A7r5 vascular smooth muscles cells and prostate cancer cells, however, is mediated by p38 MAPK and caspase-3 [20], [32]. Consistent with the latter evidence, our experiments demonstrate that AT2R overexpression induced apoptosis in HCC cells via an extrinsic cell death signaling pathway that is dependent on activation of p38 MAPK, pJNK, and caspase-3 and inactivation of pp42/44 MAPK.

One of the current study objectives is to examine the possible inhibition of human hepatocellular carcinoma tumor growth mediated by AT2R overexpression in vivo, since AT2R overexpression can induce apoptosis in HCC cells in vitro. To our surprise, moderately increasing AT2R expression actually promoted HCC tumor growth (Fig. 7A-C) and cell proliferation (Fig. 8).

With respect to the role of AngII in tumorigenesis, the involvement of AT1R has been well documented [33]–[36], the role of the AT2R in tumorigenesis remained controversial. A recent study notes that AT2R overexpression significantly attenuates the growth of fast-growing Lewis lung carcinoma (LLC) tumors, suggestive of AT2R as a potential gene target for lung cancer therapy [37]. There is also evidence for host cell AT2R deficiency-stimulated growth of murine pancreatic carcinoma grafts [38]. Conversely, the pro-oncogenic role of the AT2R has been demonstrated in carcinogen-induced colon and lung tumorigenesis in mice [39], [40], although the AT2R appears to enhance carcinogen metabolism rather than promoting cancer cell proliferation in these models. Furthermore, one recent study has described that AT2R has a key role in tumorigenesis by promoting tumor development, favoring both malignant cell proliferation and tumor angiogenesis in two different in vivo tumor models (FVB/N AT2R-KO mice in which fibrosarcomas were induced by 3-MCA injection and C57BL/6 mice with LL/2-xenografted carcinoma) [41].

In most pathophysiological situations and in some experimental models of angiogenesis [42], [43], AT2R is thought to oppose the actions of the AT1R subtype. Yet other data indicate that AT2R may be pro-angiogenic [44] and work in concert with the AT1R subtype to increase VEGF levels and blood vessel formation [45], [46]. These conflicting reports leave open the question of whether AT2R activation has beneficial or deleterious effects on tumor angiogenesis.

The apparent inconsistencies regarding AT2R may be due to differential presence and relative distribution of angiotensin receptor subtypes in in vitro and in vivo environment, or related to organ specificity of these receptors. We also contend that the AT2R receptor expression level is a major factor impacting cell proliferation, apoptosis, and that increasing AT2R expression over certain threshold may achieve inhibition of the HCC tumor growth.

We employed the typical adenoviral gene transfer approach in the current study. However, a more recent alternative, i.e., adeno-associated virus (AAV) has emerged as an attractive vector for gene therapy because of its long-term unabated gene expression, the inability to autonomously replicate without a helper virus, transduction of dividing and non dividing cells, and the lack of pathogenicity from wild-type infections [47]. A number of Phase I and Phase II clinical trials utilizing AAV have been carried out worldwide [48]–[50]. We are in the process of generating an AAV type 8 vector for inducing robust AT2R overexpression at high levels in the in vivo HCC tumor model. We hope that this approach will aid in achieving inhibition of HCC tumor growth.

In summary, we have newly discovered that while the high level AT2R overexpression in HCC cell lines inhibits proliferation and stimulates apoptosis, a moderate increased AT2R expression exerts the opposite effects in vivo, leading to promotion of the HCC tumor growth and cell proliferation. The precise mechanisms of this phenomenon are yet to be fully determined. The implications of our novel findings are two-fold, (1) AT2R overexpression may serve as potential gene transfer therapeutics for human hepatocellular carcinoma; (2) manipulating the level of AT2R overexpression may alter functional outcomes in regards to HCC tumorigenesis.

## Supporting Information

Figure S1 **AT2R overexpression induces apoptosis of Bel7402 cells. A**, Bel7402 cells were transduced with either Ad-CMV-EGFP or Ad-G-AT2R-EGFP (500 ifu/cell) for 24 hrs as described in Materials and Methods. Two representative phase-contrast micrographs from each treatment condition. Representative fluorescence micrographs from Ad-CMV-EGFP– transduced and Ad-G-AT2R-EGFP–transduced cells, showing EGFP fluorescence, TUNEL-positive (apoptotic) cell (red fluorescence nuclei), and merged (G+A) EGFP/AT2R in each treatment condition. *Scale bars*, 250 µm. **B**, quantification of the TUNEL-positive cells as a percent of the total number of cells in the dish. Columns, mean of three experiments; bars, SE. **P<0.01 versus GFP group.
(PDF)
