# Supplementary figures and images for: Effects of Angiotensin II Type 2 Receptor Overexpression on the Growth of Hepatocellular Carcinoma Cells In Vitro and In Vivo
Source: PLoS One. 2013 Dec 31;8(12):e83754. doi: 10.1371/journal.pone.0083754 (PMC3877089; doi:10.1371/journal.pone.0083754)

A

## Bel7402 Cells

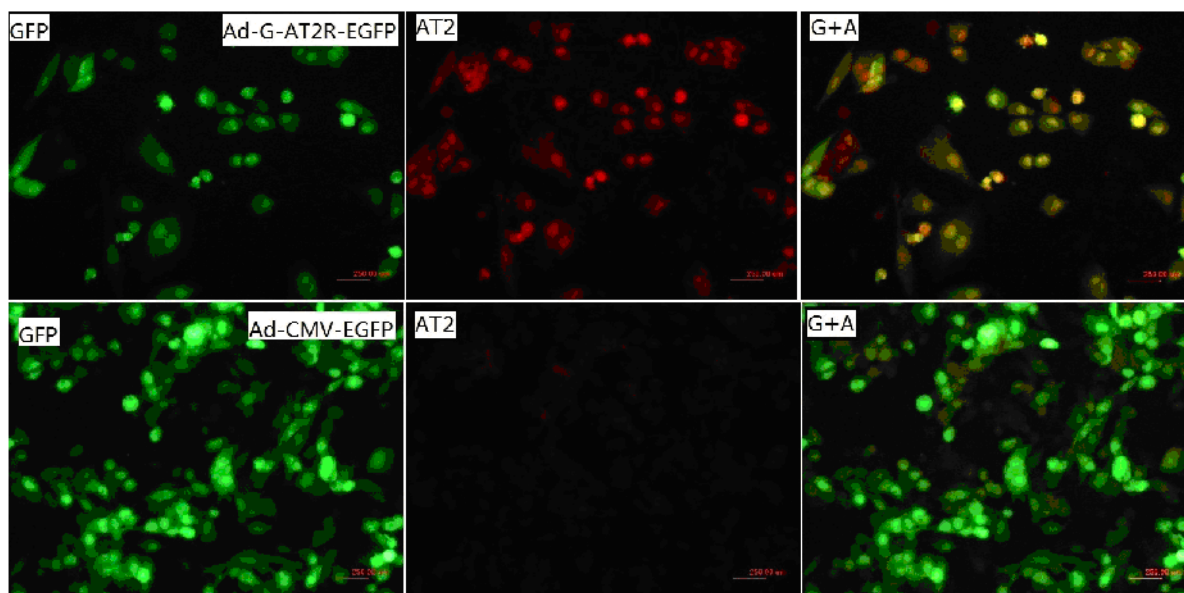

B

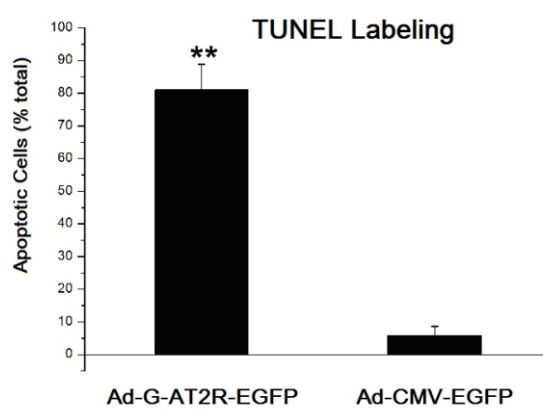

Supplement: Figure S1 — AT2R overexpression induces apoptosis of Bel7402 cells. A, Bel7402 cells were transduced with either Ad-CMV-EGFP or Ad-G-AT2R-EGFP (500 ifu/cell) for 24 hrs as described in Materials and Methods. Two representative phase-contrast micrographs from each treatment condition. Representative fluorescence micrographs from Ad-CMV-EGFP– transduced and Ad-G-AT2R-EGFP–transduced cells, showing EGFP fluorescence, TUNEL-positive (apoptotic) cell (red fluorescence nuclei), and merged (G+A) EGFP/AT2R in each treatment condition. Scale bars, 250 µm. B, quantification of the TUNEL-positive cells as a percent of the total number of cells in the dish. Columns, mean of three experiments; bars, SE. **P<0.01 versus GFP group. (PDF) [file pone.0083754.s001.pdf]
